# Supplementary material for: Human Genetic Variation Associates With Infection by Derived Ugandan M. tuberculosis Lineage
Source: J Infect Dis. 2026 Apr 18;234(3):e634–42. doi: 10.1093/infdis/jiag218 (PMC13600372; doi:10.1093/infdis/jiag218)

SUPPLEMENTAL TABLES

Supplemental Table 1. Concordance and discordance of lineages for households with two TB cases

Household number	Concordant	Lineages
90749	No	L4Uganda and L4NonUganda
91505	Yes	L3
91545	Yes	L4Uganda
92065	Yes	L4NonUganda

Supplemental Table 2. SNPs associated with L4-Uganda lineage at $p<10^{-5}$ threshold in meta-analysis

chr	Position (build 38)	rsID	Ref_allele	Alt_allele	gwasP	beta	se	MAF
1	79077303	rs61522925	C	CT	5.62E-06	-0.97469	0.214674	0.4153
1	79080689	rs4311840	A	T	6.64E-06	-0.96576	0.214381	0.4153
1	79081588	rs113330397	A	AG	3.46E-06	-0.98832	0.212934	0.4281
1	79087092	rs10874010	G	A	2.31E-06	-0.99774	0.211184	0.4281
1	79087986	rs7533890	G	A	4.64E-06	-0.97971	0.21388	0.4153
1	156736467	rs12036794	T	C	2.29E-06	1.344341	0.284471	0.1286
1	156760371	rs11264536	A	G	9.25E-08	1.448959	0.271295	0.152
1	156765602	rs11587467	A	G	1.55E-07	1.195623	0.227912	0.2965
1	156776463	rs34667125	T	TAG	7.38E-07	1.219986	0.24641	0.2179
1	156776816	rs1412310	A	G	4.90E-07	1.223313	0.243187	0.2179

1	156783065	rs10082023	T	C	7.42E-06	1.056127	0.235672	0.3404
1	156784486	rs11264551	A	G	3.78E-06	1.18531	0.256383	0.1868
1	156788545	rs56338469	C	T	1.02E-06	1.213208	0.248227	0.2179
1	156788810	rs111896519	T	C	1.03E-06	1.213089	0.248265	0.2179
1	156792798	rs11589028	C	A	9.68E-06	1.098494	0.248297	0.2247
1	241189953	rs58474067	A	T	4.62E-06	-1.42872	0.311851	0.1399
1	241189976	rs56809646	G	A	5.99E-06	-1.41313	0.312175	0.1399
1	244250228	rs56879263	A	C	7.18E-06	-1.53216	0.341368	0.1505
2	45554214	rs76565989	C	T	4.83E-06	-1.49419	0.326802	0.1551
2	50661834	rs77009079	T	C	5.00E-06	-1.42413	0.311986	0.1823
2	136453421	rs578935	C	T	6.13E-06	-1.48583	0.328593	0.09531
2	148565070	rs2034268	A	G	2.74E-06	-1.37237	0.292672	0.1634
2	148613271	rs10204718	A	G	1.93E-07	-1.52299	0.292571	0.1672
2	211316552	rs7607022	G	A	5.99E-06	-1.52759	0.337447	0.1778
2	211320435	rs73081744	A	G	6.99E-06	-1.55585	0.346205	0.1551
2	211321562	rs151103011	C	CATT	2.19E-06	-1.62669	0.343549	0.1725
2	221354899	rs6734155	A	G	3.86E-06	-1.31871	0.285495	0.2118
3	70726308	rs10658069	CAG	C	5.93E-06	-1.56746	0.34611	0.1271
4	25764088	rs28753900	A	G	9.22E-06	1.043212	0.235244	0.3238
4	27903914	rs6841769	A	C	1.44E-06	-1.10495	0.229262	0.27
4	118829390	rs13145450	C	T	8.96E-06	-1.42227	0.320267	0.1097
4	118836988	rs67882376	A	G	7.40E-06	-1.41682	0.316118	0.112
4	118841796	rs2162400	G	T	1.50E-06	-1.44062	0.299421	0.09607

4	118845233	rs34371198	C	G	2.07E-06	-1.44063	0.303514	0.09531
5	4375909	rs261129	T	C	1.37E-06	-1.3132	0.271904	0.2216
5	95056061	rs13359900	C	T	7.96E-06	-1.75766	0.393537	0.1301
5	114498966	rs3891371	G	A	2.97E-06	-1.10703	0.236892	0.4206
5	180318666	rs114797869	T	C	4.77E-06	-1.72624	0.377354	0.0885
6	110596656	rs114245171	A	G	5.67E-06	-1.40901	0.310471	0.1036
6	111621048	rs12526064	G	T	5.77E-06	-1.32601	0.292419	0.2005
6	111621101	rs12524233	A	G	5.69E-06	-1.3259	0.292195	0.2005
9	9001075	rs10977434	T	G	9.15E-06	-1.24971	0.281703	0.1884
9	70979711	rs11142658	C	A	6.73E-07	-1.26815	0.255206	0.3275
10	63638031	rs16918709	G	C	8.24E-06	-1.39373	0.312568	0.1006
10	107259302	rs56348641	T	C	6.49E-06	-1.10761	0.24561	0.2693
12	91522849	rs75164167	G	T	3.68E-06	1.400001	0.302451	0.1248
12	104612404	rs10861254	T	C	5.76E-06	-1.05373	0.232359	0.2277
12	113344819	rs141837440	A	G	5.54E-06	-1.40741	0.309773	0.118
12	113344953	rs150799822	A	G	5.57E-06	-1.4072	0.309801	0.1188
12	113345412	rs111798104	C	T	1.13E-06	-1.48579	0.305246	0.1203
12	113346021	rs112606094	G	A	1.22E-06	-1.48457	0.305932	0.1188
14	100278133	rs541153352	TA	T	3.73E-06	-1.47762	0.319436	0.1513
16	837731	rs62031079	T	C	2.17E-06	-1.4034	0.29624	0.1422
16	837945	rs142659817	T	C	2.16E-06	-1.40337	0.296231	0.1415
16	837953	rs144270616	C	A	2.16E-06	-1.40336	0.296231	0.1415
16	838512	rs74631367	A	T	2.16E-06	-1.40357	0.296266	0.1422
16	839087	rs9652787	C	T	1.93E-06	-1.40025	0.294161	0.1558
16	839248	rs9972769	C	G	2.11E-06	-1.40394	0.29601	0.1422
16	839253	rs62012366	T	C	2.08E-06	-1.39589	0.29418	0.1422

16	840155	rs62012367	A	G	7.37E-07	-1.37997	0.278712	0.1785
16	840260	rs62012368	T	C	7.42E-07	-1.38228	0.279245	0.1785
16	840308	rs62012369	G	C	7.38E-07	-1.37995	0.278712	0.1785
16	840327	rs62012370	A	G	6.77E-07	-1.37772	0.277325	0.1785
16	840525	rs62012371	T	C	4.59E-06	-1.54445	0.337002	0.1339
16	840643	rs62012372	A	G	2.15E-06	-1.39558	0.294479	0.1369
16	841006	rs62012373	T	C	2.70E-06	-1.38656	0.295503	0.1415
16	841050	rs62012374	G	A	5.32E-06	-1.32933	0.29204	0.1483
16	844345	rs62012403	G	T	9.24E-06	-1.14149	0.257422	0.2118
16	20700594	rs163254	C	T	5.96E-06	1.026428	0.226692	0.354
16	20706038	rs121564	T	G	8.89E-06	0.996751	0.224359	0.3563
16	20728999	rs4783487	T	C	9.02E-06	-0.99817	0.224842	0.3533
16	31357495	rs11574635	C	G	5.41E-06	-1.23263	0.271006	0.2201
16	31359444	rs13336235	A	G	5.48E-06	-1.23185	0.271009	0.2239
19	30250716	rs12972995	C	G	9.47E-06	1.150601	0.259787	0.1974
20	38649969	rs220522	A	T	8.92E-06	1.479049	0.332988	0.118
20	38650180	rs220521	C	T	6.14E-06	1.175569	0.25999	0.1604
20	38651713	rs4812127	G	T	9.04E-06	1.477578	0.332864	0.1188
20	38666240	rs220503	T	C	5.93E-06	1.41575	0.312595	0.1331
20	38669217	rs220498	T	C	6.08E-06	1.41587	0.313008	0.1316
21	21626716	rs1999296	T	C	7.29E-06	-1.09354	0.243822	0.2844
22	50573113	rs17001616	A	C	8.26E-06	-1.3952	0.312943	0.2088

Supplemental Table 3. Results for top SNPs separately by cohort

	Cohort 1		Cohort 2		Meta analysis	
SNPID	Est_Cohort1	P-Value_Cohort1	Est_Cohort2	p-value Cohort2	beta	pval
1:79077303	-1.09793	5.80E-05	-0.78498	2.15E-02	-0.97469	5.62E-06
1:79080689	-1.08169	7.22E-05	-0.78634	2.13E-02	-0.96576	6.64E-06
1:79081588	-1.05656	8.81E-05	-0.87981	1.01E-02	-0.98832	3.46E-06
1:79087092	-1.05783	7.23E-05	-0.90097	8.11E-03	-0.99774	2.31E-06
1:79087986	-1.08910	6.16E-05	-0.81025	1.74E-02	-0.97971	4.64E-06
1:156736467	1.08688	6.02E-03	1.61483	5.17E-05	1.34434	2.29E-06
1:156760371	1.40514	4.33E-04	1.48642	4.40E-05	1.44896	9.25E-08
1:156765602	1.02660	9.28E-04	1.38995	2.54E-05	1.19562	1.55E-07
1:156776463	1.07587	1.74E-03	1.37088	7.78E-05	1.21999	7.38E-07
1:156776816	1.08867	1.22E-03	1.36843	7.24E-05	1.22331	4.90E-07
1:156783065	0.92488	3.08E-03	1.22611	5.28E-04	1.05613	7.42E-06
1:156784486	1.00770	4.01E-03	1.38682	1.71E-04	1.18531	3.78E-06
1:156788545	1.08529	1.66E-03	1.34882	1.22E-04	1.21321	1.02E-06
1:156788810	1.08500	1.67E-03	1.34879	1.22E-04	1.21309	1.03E-06
1:156792798	1.00534	3.36E-03	1.19967	7.23E-04	1.09849	9.68E-06
1:241189953	-1.21193	4.07E-03	-1.68572	2.08E-04	-1.42872	4.62E-06
1:241189976	-1.20984	4.19E-03	-1.65377	2.74E-04	-1.41313	5.99E-06
1:244250228	-1.76573	3.12E-04	-1.30944	6.09E-03	-1.53216	7.18E-06
2:45554214	-1.36917	1.16E-03	-1.68085	1.20E-03	-1.49419	4.83E-06
2:50661834	-1.61180	1.89E-05	-0.98533	8.39E-02	-1.42413	5.00E-06
3:70726308	-1.33534	2.10E-03	-1.95508	6.72E-04	-1.56746	5.93E-06
2:136453421	-1.24901	3.89E-03	-1.79351	2.91E-04	-1.48583	6.13E-06
2:148565070	-1.50752	8.16E-05	-1.18978	7.90E-03	-1.37237	2.74E-06
2:148613271	-1.79056	2.80E-06	-1.15771	9.95E-03	-1.52299	1.93E-07

2:211316552	-1.69480	1.87E-04	-1.31860	8.98E-03	-1.52759	5.99E-06
2:211320435	-1.80373	1.35E-04	-1.26479	1.29E-02	-1.55585	6.99E-06
2:211321562	-1.88949	5.85E-05	-1.31843	9.03E-03	-1.62669	2.19E-06
2:221354899	-1.70808	4.66E-06	-0.78394	7.47E-02	-1.31871	3.86E-06
4:25764088	0.95027	1.92E-03	1.17361	1.22E-03	1.04321	9.22E-06
4:27903914	-0.76555	1.35E-02	-1.50984	7.96E-06	-1.10495	1.44E-06
4:118829390	-1.73655	3.13E-05	-0.95425	5.90E-02	-1.42227	8.96E-06
4:118836988	-1.68421	3.87E-05	-1.00011	4.79E-02	-1.41682	7.40E-06
4:118841796	-1.69889	1.44E-05	-1.03637	2.98E-02	-1.44062	1.50E-06
4:118845233	-1.71341	1.72E-05	-1.02352	3.31E-02	-1.44063	2.07E-06
5:4375909	-1.25912	3.13E-04	-1.39388	1.05E-03	-1.31320	1.37E-06
5:95056061	-0.93262	1.06E-01	-2.47794	9.62E-06	-1.75766	7.96E-06
5:114498966	-0.89460	2.65E-03	-1.46480	1.34E-04	-1.10703	2.97E-06
5:180318666	-1.62011	1.26E-03	-1.86259	1.08E-03	-1.72624	4.77E-06
6:110596656	-1.47594	5.30E-04	-1.33431	3.33E-03	-1.40901	5.67E-06
6:111621048	-1.25013	9.46E-04	-1.43636	1.58E-03	-1.32601	5.77E-06
6:111621101	-1.25013	9.46E-04	-1.43567	1.56E-03	-1.32590	5.69E-06
9:70979711	-1.11160	2.33E-03	-1.41494	6.31E-05	-1.26815	6.73E-07
9:9001075	-1.11843	2.57E-03	-1.41982	8.12E-04	-1.24971	9.15E-06
10:63638031	-1.85924	9.36E-06	-0.80404	8.75E-02	-1.39373	8.24E-06
10:107259302	-1.03725	7.96E-04	-1.22476	2.23E-03	-1.10761	6.49E-06
12:104612404	-0.97364	1.80E-03	-1.15088	7.99E-04	-1.05373	5.76E-06
12:113344819	-1.53210	5.83E-04	-1.29117	2.58E-03	-1.40741	5.54E-06
12:113344953	-1.53202	5.85E-04	-1.29084	2.59E-03	-1.40720	5.57E-06
12:113345412	-1.76306	5.70E-05	-1.21883	4.18E-03	-1.48579	1.13E-06
12:113346021	-1.76179	6.31E-05	-1.22082	4.10E-03	-1.48457	1.22E-06
12:91522849	1.22603	1.82E-03	1.63818	4.15E-04	1.40000	3.68E-06
14:100278133	-1.61438	1.58E-04	-1.30070	6.34E-03	-1.47762	3.73E-06

16:20700594	0.86326	4.78E-03	1.22181	2.49E-04	1.02643	5.96E-06
16:20706038	0.81423	6.72E-03	1.22371	2.43E-04	0.99675	8.89E-06
16:20728999	-0.81775	6.14E-03	-1.23130	2.62E-04	-0.99817	9.02E-06
16:31357495	-1.50059	2.37E-05	-0.84305	4.71E-02	-1.23263	5.41E-06
16:31359444	-1.49942	2.49E-05	-0.84629	4.56E-02	-1.23185	5.48E-06
16:837731	-1.22497	1.40E-03	-1.65981	1.99E-04	-1.40340	2.17E-06
16:837945	-1.22494	1.40E-03	-1.65981	1.99E-04	-1.40337	2.16E-06
16:837953	-1.22494	1.40E-03	-1.65978	1.99E-04	-1.40336	2.16E-06
16:838512	-1.22487	1.40E-03	-1.66044	1.98E-04	-1.40357	2.16E-06
16:839087	-1.22677	1.36E-03	-1.64222	1.79E-04	-1.40025	1.93E-06
16:839248	-1.22732	1.37E-03	-1.65670	1.98E-04	-1.40394	2.11E-06
16:839253	-1.21744	1.34E-03	-1.65670	1.98E-04	-1.39589	2.08E-06
16:840155	-1.13687	1.62E-03	-1.73104	4.90E-05	-1.37997	7.37E-07
16:840260	-1.13687	1.62E-03	-1.74002	4.94E-05	-1.38228	7.42E-07
16:840308	-1.13682	1.62E-03	-1.73104	4.90E-05	-1.37995	7.38E-07
16:840327	-1.13682	1.62E-03	-1.71729	4.42E-05	-1.37772	6.77E-07
16:840525	-1.40242	1.70E-03	-1.72413	4.76E-04	-1.54445	4.59E-06
16:840643	-1.21633	1.37E-03	-1.65676	1.98E-04	-1.39558	2.15E-06
16:841006	-1.22740	1.38E-03	-1.61161	2.77E-04	-1.38656	2.70E-06
16:841050	-1.22973	1.35E-03	-1.46277	7.25E-04	-1.32933	5.32E-06
16:844345	-1.11534	1.09E-03	-1.17561	2.44E-03	-1.14149	9.24E-06
19:30250716	1.03780	1.15E-03	1.36833	1.86E-03	1.15060	9.47E-06
20:38649969	1.31461	3.76E-03	1.66148	4.85E-04	1.47905	8.92E-06
20:38650180	1.19587	7.87E-04	1.15340	2.02E-03	1.17557	6.14E-06
20:38651713	1.31445	3.76E-03	1.65827	4.94E-04	1.47758	9.04E-06
20:38666240	1.33846	2.55E-03	1.48843	4.89E-04	1.41575	5.93E-06
20:38669217	1.33599	2.62E-03	1.49120	4.89E-04	1.41587	6.08E-06
21:21626716	-1.00989	1.57E-03	-1.20780	1.25E-03	-1.09354	7.29E-06

22:50573113	-1.13730	6.53E-03	-1.71854	2.42E-04	-1.39520	8.26E-06

Supplemental Table 4. Sensitivity analysis including only the index case per household

SNPID	Beta	pvalue
1:79077303	-0.96165	8.54E-06
1:79080689	-0.95265	1.01E-05
1:79081588	-0.97633	5.25E-06
1:79087092	-0.98548	3.61E-06
1:79087986	-0.96614	7.25E-06
1:156736467	1.31189	5.24E-06
1:156760371	1.41436	2.69E-07
1:156765602	1.15985	5.07E-07
1:156776463	1.18058	2.30E-06
1:156776816	1.18487	1.54E-06
1:156783065	1.06207	7.31E-06
1:156784486	1.14896	9.66E-06
1:156788545	1.17291	3.16E-06
1:156788810	1.17278	3.18E-06
1:156792798	1.05679	2.79E-05
1:241189953	-1.45188	3.43E-06
1:241189976	-1.43480	4.56E-06
1:244250228	-1.53185	9.01E-06
2:45554214	-1.55911	2.20E-06
2:50661834	-1.44143	3.97E-06
2:136453421	-1.55900	2.63E-06
2:148565070	-1.35433	5.08E-06

2:148613271	-1.51522	3.26E-07
2:211316552	-1.59996	2.55E-06
2:211320435	-1.63186	2.96E-06
2:211321562	-1.70340	8.76E-07
2:221354899	-1.26892	1.12E-05
3:70726308	-1.60842	3.85E-06
4:25764088	1.01878	1.89E-05
4:27903914	-1.13063	1.05E-06
4:118829390	-1.40688	1.36E-05
4:118836988	-1.40188	1.12E-05
4:118841796	-1.43073	2.17E-06
4:118845233	-1.43033	3.00E-06
5:4375909	-1.28287	4.49E-06
5:95056061	-1.80919	7.09E-06
5:114498966	-1.08556	5.85E-06
5:180318666	-1.82681	1.72E-06
6:110596656	-1.41133	7.28E-06
6:111621048	-1.38419	2.56E-06
6:111621101	-1.38433	2.52E-06
9:70979711	-1.24628	1.29E-06
9:9001075	-1.26226	9.32E-06
10:63638031	-1.38026	1.18E-05
10:107259302	-1.13833	4.78E-06
12:91522849	1.37385	6.99E-06
12:104612404	-1.01485	1.63E-05
12:113344819	-1.39724	8.33E-06
12:113344953	-1.39701	8.37E-06
12:113345412	-1.47661	1.75E-06

12:113346021	-1.47514	1.89E-06
14:100278133	-1.42673	1.14E-05
16:837731	-1.40994	4.67E-06
16:837945	-1.40991	4.67E-06
16:837953	-1.40990	4.67E-06
16:838512	-1.41013	4.67E-06
16:839087	-1.40720	4.18E-06
16:839248	-1.41052	4.55E-06
16:839253	-1.40175	4.50E-06
16:840155	-1.38933	1.30E-06
16:840260	-1.38933	1.30E-06
16:840308	-1.38930	1.30E-06
16:840327	-1.38733	1.19E-06
16:840525	-1.56661	1.00E-05
16:840643	-1.40143	4.64E-06
16:841006	-1.39350	5.70E-06
16:841050	-1.33062	1.11E-05
16:844345	-1.14990	9.63E-06
16:20700594	1.07928	2.82E-06
16:20706038	1.04756	4.34E-06
16:20728999	-1.05013	4.27E-06
16:31357495	-1.20972	1.03E-05
16:31359444	-1.20802	1.06E-05
19:30250716	1.13146	1.44E-05
20:38649969	1.52835	6.63E-06
20:38650180	1.20544	4.57E-06
20:38651713	1.52665	6.73E-06
20:38666240	1.42460	1.04E-05

20:38669217	1.42353	1.08E-05
21:21626716	-1.11149	6.03E-06
22:50573113	-1.37349	1.57E-05

Supplemental Table 5. HIV-only negative

SNPID	beta	pvalue
1:79077303	-1.04788	8.47E-06
1:79080689	-1.03513	1.15E-05
1:79081588	-1.08786	3.14E-06
1:79087092	-1.09872	1.99E-06
1:79087986	-1.05346	7.43E-06
1:156736467	1.16004	4.85E-04
1:156760371	1.43318	7.86E-06
1:156765602	1.15724	5.44E-06
1:156776463	1.28930	6.93E-06
1:156776816	1.30247	4.76E-06
1:156783065	1.07011	6.62E-05
1:156784486	1.19397	4.79E-05
1:156788545	1.30150	6.60E-06
1:156788810	1.30111	6.66E-06
1:156792798	1.14629	5.75E-05
1:241189953	-1.42011	1.05E-04
1:241189976	-1.41988	1.07E-04
1:244250228	-1.37652	2.06E-04
2:45554214	-1.37188	1.77E-04
2:50661834	-1.58505	4.47E-06

2:136453421	-1.22925	1.07E-03
2:148565070	-1.45565	4.05E-06
2:148613271	-1.54364	8.97E-07
2:211316552	-1.75526	2.68E-06
2:211320435	-1.82431	2.02E-06
2:211321562	-1.88772	7.28E-07
2:221354899	-1.31713	2.44E-05
3:70726308	-1.65028	1.54E-05
4:25764088	1.04115	5.94E-05
4:27903914	-1.34850	1.10E-07
4:118829390	-1.43613	1.13E-04
4:118836988	-1.40663	1.17E-04
4:118841796	-1.31571	1.79E-04
4:118845233	-1.29125	2.94E-04
5:4375909	-1.11754	2.85E-04
5:95056061	-1.37050	1.81E-03
5:114498966	-1.01332	7.39E-05
5:180318666	-1.48326	4.19E-04
6:110596656	-1.46115	1.01E-05
6:111621048	-1.45518	7.77E-06
6:111621101	-1.45485	7.64E-06
9:70979711	-1.26374	1.42E-05
9:9001075	-1.12083	3.94E-04
10:63638031	-1.38813	6.82E-05
10:107259302	-1.09586	5.12E-05
12:91522849	1.40196	3.38E-05
12:104612404	-0.98877	2.17E-04
12:113344819	-1.24613	4.06E-04

12:113344953	-1.25979	3.64E-04
12:113345412	-1.29799	1.89E-04
12:113346021	-1.29577	2.03E-04
14:100278133	-1.44654	8.43E-05
16:837731	-1.63938	1.04E-06
16:837945	-1.63534	1.04E-06
16:837953	-1.63532	1.04E-06
16:838512	-1.63576	1.04E-06
16:839087	-1.56179	1.91E-06
16:839248	-1.63488	1.02E-06
16:839253	-1.58991	1.44E-06
16:840155	-1.63509	4.56E-07
16:840260	-1.63509	4.56E-07
16:840308	-1.63525	4.55E-07
16:840327	-1.63814	3.96E-07
16:840525	-1.70726	6.70E-06
16:840643	-1.63802	9.89E-07
16:841006	-1.60810	1.42E-06
16:841050	-1.56682	1.80E-06
16:844345	-1.44927	1.30E-06
16:20700594	0.92345	2.47E-04
16:20706038	0.92607	2.15E-04
16:20728999	-0.92365	2.45E-04
16:31357495	-1.41417	2.68E-06
16:31359444	-1.41154	2.79E-06
19:30250716	0.99777	7.11E-04
20:38649969	1.45573	1.99E-04
20:38650180	1.18487	9.12E-05

20:38651713	1.45388	2.01E-04
20:38666240	1.57539	2.49E-05
20:38669217	1.57782	2.26E-05
21:21626716	-1.20164	8.14E-06
22:50573113	-1.21722	2.10E-04

Supplemental Table 6. Results from Selection Analysis

uniqID	Esan in Nigeria	Yoruba in Nigeria	Luhya in Kenya	Mende in Sierra Leone	Gambian Western Division	African Caribbean Barbados	African Ancestry Southwest
12:113782624:A:G	x	x				x	x
12:113782758:A:G	x	x				x	x
12:113783217:C:T	x	x	x	x		x	x
12:113783826:A:G	x	x	x	x		x	

Supplemental Table 7. Results from eQTL query

gene	snp_hg38	pval
RRNAD1	chr1:156792798:A:C	0.022
RRNAD1	chr1:156788810:C:T	0.03
RRNAD1	chr1:156736467:C:T	0.036
NARFL	chr16:837731:C:T	0.045
NARFL	chr16:837945:C:T	0.045
NARFL	chr16:837953:A:C	0.045
NARFL	chr16:839253:C:T	0.045
NARFL	chr16:840525:C:T	0.045
NARFL	chr16:841006:C:T	0.045
NARFL	chr16:841050:A:G	0.045
NARFL	chr16:840260:C:T	0.048
NARFL	chr16:840308:C:G	0.048

Supplemental Table 8 – TB candidate gene results

	Total SNPs	SNPs with P-value < 0.05	%of total SNPs	Most Sig P-value
CD209	87	1	1.15	0.0217
HLA-A	278			
HLA-DQB1	299			
HLA-DRB1	145			
IFNG	22	1	4.55	0.0471
IFNG-AS1	558	11	1.97	0.0048
IFNGR1	18			
IL10	23			
MBL2	49	9	18.37	0.0038
MCP1	20			
SLC11A1	42	8	19.05	0.0055
TLR2	52	3	5.77	0.0043
TLR4	59	6	10.17	0.0189
TLR9	13	2	15.38	0.0331
TNFA	19	1	5.26	0.0285
VDR	249	13	5.22	0.0070

Supplemental Table 9 – Previously studied host-pathogen interaction effects in TB

Gene	Paper ref	Study population	Associated lineage	# SNPs/total SNPs p<0.05	most sig p-val
MARCO	Thuong	Vietnam	Beijing	0.0000	0.0780
TLR2	Caws	Vietnam	Beijing	0.0857	0.0043
FLOT1	Luo	Peru	L2	0.0000	0.0608
CD53	Omae	Thailand	Beijing	0.5479	0.00003
HLA-B	Salie	South Africa	Beijing	0.0000	0.1090
HLA-B	Salie	South Africa	Beijing	0.2736	0.0006
HLA-B	Toyo-oka	Thailand	"Modern"	0.0000	0.0588
SLC11A1	van Crevel	Indonesia	Beijing	0.2800	0.0055
IRGM	Intermann	Ghana	Mtb vs M africanum	0.0000	0.0623
LAMP1	Songame	Indonesia	Beijing	0.0000	0.2114
MTOR	Songame	Indonesia	Beijing	0.0000	0.0784
DAP	Phelan	Thailand	L2.2	0.0000	0.0906
DAP	Phelan	Thailand	L2.2	0.0000	0.1572
chr14intergenic	Phelan	Thailand	L4.4	0.0556	0.0400
MFAP2	Phelan	Thailand	L2.2	0.0000	0.0774
MFAP2	Phelan	Thailand	L2.2	0.0000	0.2247
FSTL5	Phelan	Thailand	L2.2	0.0397	0.0036
FSTL5	Phelan	Thailand	L2.2	0.1000	0.0015
chr2intergenic	Phelan	Thailand	L1.1	0.0000	0.1378
RIMS3	Phelan	Thailand	L1.1	0.0000	0.1148
RIMS3	Phelan	Thailand	L1.1	0.6000	0.0077
chr3intergenic	Phelan	Thailand	L4.4	0.0000	0.1619
CSGALNACT1	Phelan	Thailand	L2.2	0.0127	0.0061
CSGALNACT1	Phelan	Thailand	L2.2	0.0000	0.1673

Supplemental Figure 1. Correlation between GWAS findings for L4-Uganda vs L4-NonUganda (x-axis) and L4-Uganda vs all other lineages (y-axis), for results in primary GWAS where p < 0.005

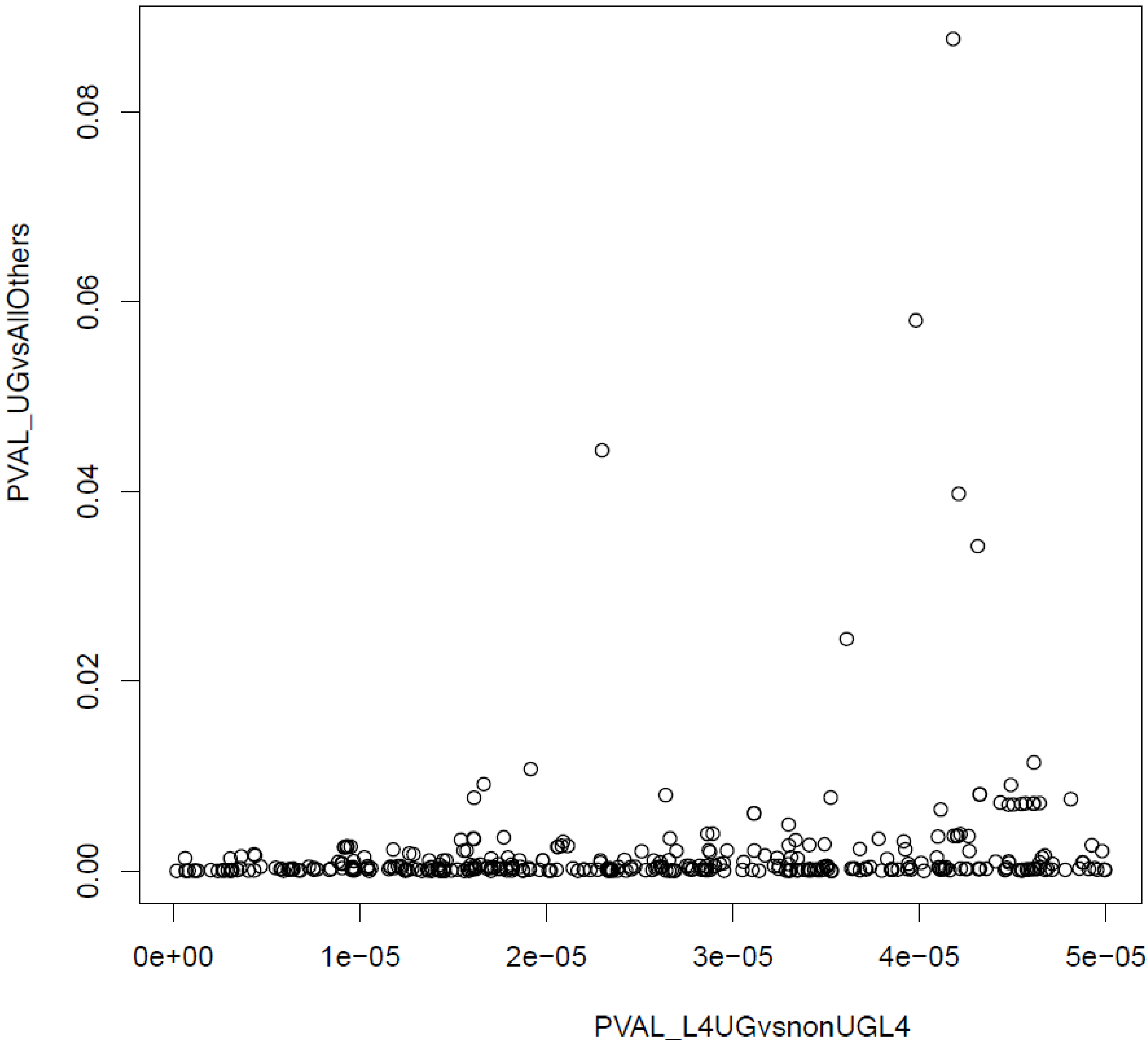

Supplement: Supplementary file 1 [file jiag218_supplementary_data.pdf]
